# Sequelae after AF ablation: Efficacy and Safety go Hand in Hand

**DOI:** 10.1016/s0972-6292(16)30523-x

**Published:** 2012-07-28

**Authors:** Yves De Greef, R Tavernier, M Duytschaever

**Affiliations:** 1 Department of Cardiology, Antwerp Cardiovascular Institute Middelheim, Belgium; 2 Department of Cardiology, Sint Jan Hospital Bruges, Belgium; 3 University Hospital of Ghent, Belgium

**Keywords:** Ablation, atrial fibrillation, complications, efficacy, sequellae, pulmonary vein isolation

## Abstract

Although nowadays performed on a routine basis, catheter ablation of atrial fibrillation is associated with the potential for major complications. Improving the safety remains therefore an important challenge. This article summarizes the different types of complications associated with AF ablation grouping them into clinically overt major complications, subclinical injury and permanent injury. Furthermore, it describes the potential predictors for complications and highlights the dynamic interplay between efficacy and safety.

## Major complications and sequellae after ablation of AF

Percutaneous catheter ablation is a well-established, efficient and effective treatment strategy for recurrent, symptomatic and drug-resistant atrial fibrillation (AF).[1] Major complications though occur in up to 5.9% of procedures (Table 1).[2-13] Taking into account the relatively benign nature of AF, this complication rate is a major point of concern. Besides the clinically overt major complications, one should also consider other sequellae, like ablation-induced subclinical events and permanent injury.

### Clinically overt major complications

From its early phase on, catheter ablation of AF is linked to potentially devastating complications as stroke, cardiac tamponade, atrio-esophageal fistula and pulmonary vein (PV) stenosis. Furthermore death might occur in up to 1 out of 1000 patients.[14] Table 1 lists up the prevalence of clinically overt major complicationsas observed in large single-centre studies, multi-centre trials, and the world-wide surveys.[2-13] In general, a major complication - defined as any procedure related adverseevent requiring intervention, causing long-term disability or death or resulting in prolongation of hospital stay - occurs in 0.8 to 5.9% (median 4%) of procedures. Although the relative distribution of specific types of complications is different between the reports, in general there are three major "1%" complications: stroke or TIA, tamponade and major vascular injury. A wide variety of other complications accounts for the remaining 1% (Table 1). Interestingly, overall major complication rate does not differ between older and more recent reports (from the left to the right side of the table). This finding is paralleled by the similar complications rates reported in the first and second survey.[2,10]

### Subclinical events

Because of the silent nature of certain events, overall complication rate of AF ablation (as listed in Table 1) is likely to be underestimated. It is well-recognised that the prevalence of events like deep venous thrombosis, pulmonary embolism, arterio-venous fistula and PV narrowing is dependent on the scrutiny of detection. Only recently it was recognized that an overt thromboembolic event (stroke or TIA) most likely represents only a small part of the wider spectrum of cardio-embolic events. MRI studies of the brain demonstrated asymptomatic cerebral emboli (ACE) in a substantial number (14%) of patients after AF ablation.[15] Although none of the most widely-used ablation strategies seems immune to ACE, there is a variability in the incidence: use of the PVAC catheter was associated with a significantly higher rate of ACE (37.5%) in comparison to irrigated-RF (7.4%) or cryoballoon energy (4.3%).[16] Several questions regarding ACE (its clinical significance?, exact nature of the lesions seen on MRI?, reversibility?) remain unanswered and prevent us to draw more definite conclusions.

Also ablation-induced PV narrowing is an event likely to remain largely undetecteddue to its silent nature and inconsistent follow-up. Our group recently reported the impact of PVAC-ablation on PV diameters.[17] Moderate PV narrowing (i.e. diameter reduction 25% to 50%) and severe PV narrowing (i.e. diameter reduction >50%) occurred in respectively 28% and 4% of PVs.[17] Only 1 out of 50 patients showing moderate to severe PV narrowing was symptomatic. In comparison, after point-by-point circumferential PV-antrum isolation, severe PV narrowing (>50%) occurred in 3.8% of PVs.[18]

### Permanent injury

Major complications after AF ablation only rarely lead to permanent injury. Hoyt el al. reported an overall major complication rate of 4.7% but permanent deficit was observed in only 0.5% of patients.[19] Likewise, Dagres et al., reporting safety of AF ablation in 1000 procedures, stated that the vast majority of encountered major complications could be treated conservatively without permanent residues.[8] On the other hand, permanent injury might remain undetected. Schwartz et al. noted that ablated patients showed a worse neuropsychological outcome in verbal memory in comparison to baseline and control non-AF patients.[20] Although these data differ from those reported by Bunch et al.[21], it is tempting to speculate that ACE play a role in the decline of cognitive functioning. Also PV narrowing might lead to permanent sequellae. Although Arentz et al, in 11 patients with initial PV narrowing (>70% diameter reduction), observed no progression of PV stenosis at long-term follow-up (>2 years), about 63% of these patients developed pulmonary hypertension during exercise.[22] Finally, AF ablation is associated with a marked radiation exposure to the patient.[23,24] Delayed effects of radiation (cancer, genetic abnormalities) are therefore another potential cause for permanent injury. In fact, Lickfett et al. reported an additional lifetime risk for a fatal malignancy associated with AF ablation of 0.15% for female patients and 0.21% for male patients.[24,25]

## Predictors of Complications: "Efficacy and Safety go hand in hand"

**Figure 1:**
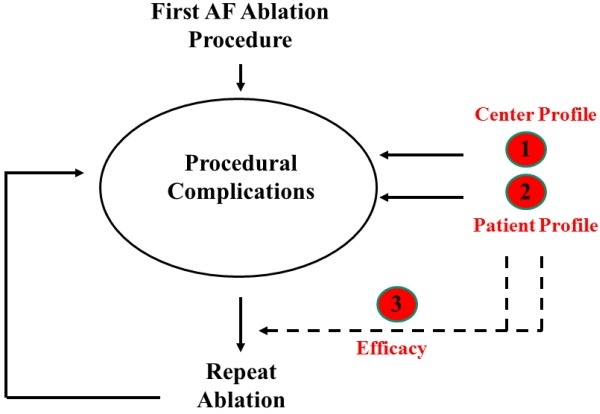
There a three major predictors of complications in AF ablation. Center profile refers to procedure volume, operator experience and interaction between nurses, fellows, anesthesiologists and electrophysiologists. The patient profile is another important predictor for complications with a higher reported procedural risk in certain subgroups (high CHADS2, female gender, older age, impaired left ventricular function). Finally, efficacy itself is a critical predictor for complications ("cumulative complication rate"): if efficacy is low and the patient needs to undergo a repeat ablation then the patient is exposed again to the same procedural risk ("safety and efficacy go hand in hand"). Interestingly efficacy itself is determined by center and patient profile (dashed lines).

To reduce and potentially avoid the overall complication rate in AF ablation, it is essential to acknowledge the predictors of complications and the dynamic interplay between efficacy and safety (Figure 1). Like in most medical procedures, center and patient profile act as key predictors of procedural complications. Interestingly, center and patient profile also determine the efficacy of AF ablation and the subsequent need for repeat ablation (dashed lines). Efficacy itself is the 3rd predictor of complications, because patients have to undergo the risk of procedural complications for a second time during a repeat procedure (cumulative complication rate).

### Center profile

It is well established that institutional experience and center volume have an impact on efficacy of AF ablation (Figure 1, dashed line).[2] More recently, center profile was found to be linked with safety as well (Figure 1, solid line). In the study by Hoyt et al., studying major complication rate over time, a significant decrease was noted from 11.1% in 2002 to1.6% in 2010 while the annual procedure volume and the number of AF ablationists increased.[19] Interestingly, center profile has a greater impact on overall procedural safety than the individual experience of the operator. In the study of Dagres et al. no relationship was found between the operator and the complication rate with a very experienced operator having the highest complication rate.[8] Hoyt et al found no statistical difference in complication rate between junior faculty (performing<30 AF ablations) and the senior faculty (>30 ablations).[19] Most likely, choices to change practice pattern over time have a major influence on complication rate. For instance, after the introduction of an esophageal temperature probe no A-E fistula was observed in the Leipzig experience.[8] Also changes in ablation strategy (wide circumferential PV isolation instead of segmental PV isolation), use of irrigated catheters or adoption of new peri-procedural anticoagulation regimens reflect center rather than operator profile.

### Patient profile

In young paroxysmal AF patients without structural heart disease and no LA enlargement, reported procedural risk can be as low as 0.8%.[12] Other studies, reporting ablation outcome in a more heterogeneous patient population, have identified subgroups at higher risk for complications: (1) a higher CHADS2 score, (2) female gender, (3) older age and (4) the presence of heart failure. Chao et al. observed that the overall procedural complication rate was greater in patients with a higher CHADS2 score.[26,27] Similarly a CHADS2 score of =2 has previously been recognised as an independent predictor of peri-procedural stroke,[28] whereas other studies reported absence of stroke in CHADS2 0 or 1 patients.[29] Patel et al. reported that female patients had more major complications (5%) compared to male patients (2.4%).[30] A higher procedural risk in women was also observed in other cardiovascular interventions like percutaneous coronary intervention.[31] Zado et al. compared AF ablation between age groups (<65yrs, 65-74y, =75y) and found a trend towards more complications in the elderly.[32] Likewise, Spragg et al reported a nearly 4-fold increase (in comparison to younger patients) in major complication rate over the age of 70.[6] Finally, Chen et al compared AF ablation in patients with normal and impaired systolic function.[33] In comparison to patients with a normal systolic function, major complication rate was higher in patients with impaired systolic function (4.3% vs 3.5%).

**Figure 2:**
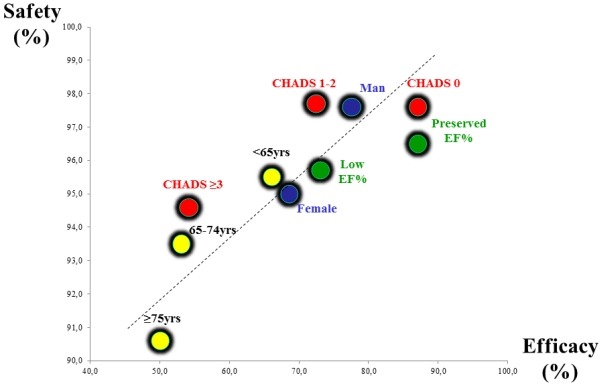
The reported risk factors (patient profile) for complications - i.e. higher CHADS2 score, female gender, older age (≥75years) and low ejection fraction -also determine the efficacy of the procedure ("efficacy and safety go hand in hand"). See text for explanation. Safety is defined as the absence of reported major complications. Efficacy is defined as freedom of AF after the first procedure with or without antiarrhythmic drugs. Red dots = study by Chao et al in subgroups with different CHADS2-scores; blue dots = study by Patel et al, in male and female subgroups; yellow dots = study by Zado et al comparing different age groups; green dots = study by Chen et al in patients with a preserved and low ejection fraction.

Interestingly, the above mentioned risk factors for complications - i.e. higher CHADS2 score, female gender, older age (=75yrs) and low ejection fraction -also determine the efficacy of the procedure ("efficacy and safety go hand in hand"). Outcome data from those studies reporting both efficacy and safety within different subgroups are plotted in Figure 2. Clearly, there is a linear relation between efficacy and safety within subgroups of each study (avoiding confounding variables like follow-up, center profile, ablation strategy etc) but also within all reported studies (dashed line). This high correlation implies that in patients at risk for a higher complication rate, also efficacy is expected to be lower ("efficacy and safety go hand in hand").

### Efficacy and cumulative complication rate

Efficacy-dependent complications are those complications related to a repeat ablation (Figure 1). The patient is again subjected to all reported complications leading to a higher "cumulative complication" rate. Although procedure time and number of lesions during repeat ablation might be limited, some complications might be more frequent compared to the first procedure e.g. due to hampered groin or transseptal access.[7] The concept of "cumulative complication rate" has several implications: (1) Complication rate in AF ablation should be reported per patient rather than per procedure. Cumulative complication rate per patient should be used to weigh the risks and benefits of a planned procedure. (2) In patients with an expected higher initial complication rate (due to patient profile), the cumulative complication rate is expected to be even much higher due to the inherent lower efficacy and higher need for a repeat procedure ('efficacy and safety go hand in hand').

## How to avoid Complications in AF Ablation?

### Novel technology

It might be a realistic goal to make AF ablation as effective and safe as other cardiac procedures, such as percutaneous coronary interventions or other ablation procedures. In a continuous quest for a more effective, faster and safer ablation approach, alternative forms of energy (ultrasound, cryo-energy, laser, microwave, etc.) and several new ablation catheters (e.g. magnetic catheters, "single-shot" devices as the cryoballoon and multi-electrode catheters) have been studied. However, it is unlikely that changes in AF ablation technology will completely eliminate procedure-related complications. Even in experienced hands, this technique carries a risk of embolic events (thrombus, air), cardiac tamponade and groin complications, all of them to some extent related to the invasive nature of the procedure (sheaths, guidewires, manipulation, electrical cardioversions) and its peri-operative changes in anticoagulation.

### Benefit-risk ratio

**Figure 3:**
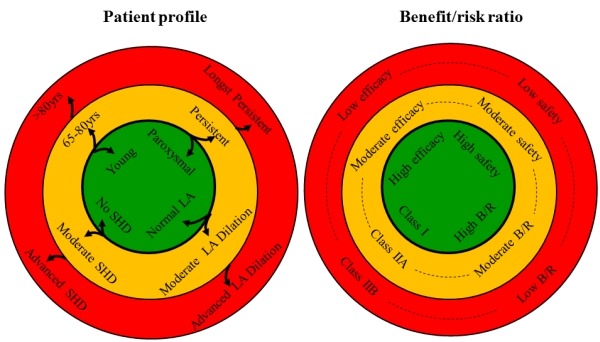
Indications (with level of recommendation) for AF ablation based upon safety and efficacy. See text for explanation. LA=Left Atrium, SHD= Structural Heart Disease, yrs=years, B/R=Benefit/Risk ratio.

Therefore it will remain essential to determine a tailored benefit/risk ratio in each individual patient referred for AF ablation. Indications for AF ablation should be based both upon safety and efficacy for each patient profile (Figure 3). In young patients with paroxysmal, lone AF (inner circle, green, left panel), efficacy and safety of AF ablation were shown to be high in randomised controlled trials, resulting in a high benefit to risk ratio and a Class I indication (right panel). In older patients, persistent AF, moderate LA enlargement (<50mm) and/or moderate structural heart disease, often undergoing AF ablation in world-wide practice (orange circle, left panel), large single-centre studies have shown that efficacy, safety and thus the benefit to risk ratio are expected to be moderate (Class IIA indication, right panel). The outer circle (red) represents patients with advanced structural remodelling: octogenarians, longstanding persistent AF, advanced LA enlargement (>50mm) and advanced structural heart disease (like CHADS 3 or more, severe valvular heart disease, severely impaired EF). Several small studies have shown that efficacy, safety and thus benefit to risk ratio are low corresponding to a Class IIB indication, right panel). This would imply a high threshold (for both symptoms and number of failed drugs) before opting for AF ablation in these patients.

### Maximising the first pass efficacy

Although operator/institutional experience and patient selection might be the crucial ways to reduce inherent procedural-related complications, maximising the first pass efficacy rate is another necessary step to avoid complications and permanent sequelae in AF ablation. Given an ablation strategy with a 4% complication rate, reducing the redo rate from 40% to 10% would decrease the cumulative complication rate from 5.6% to 4.4% (21% relative risk reduction). Given the "AF recurrence equals pulmonary vein reconnection" paradigm, any method to improve permanent pulmonary vein isolation will decrease the number of repeat procedures and as such its cumulative complication rate ("efficacy and safety go hand in hand").

## Figures and Tables

**Table 1:**
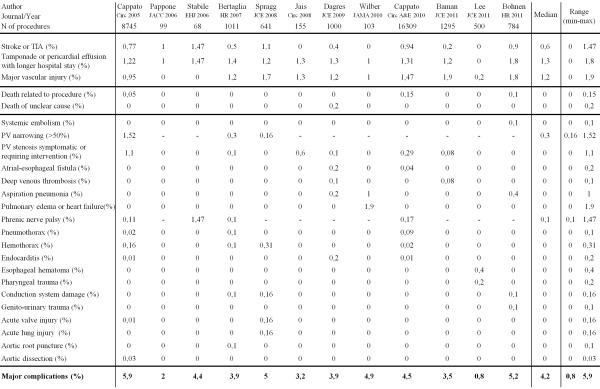
Major AF ablation reports (ranked in chronological order, from 2005 to 2011) addressing safety. See text for further explanation. N=Number, TIA=Transient Ischemic Attack.
